# Complete Genetic Analysis of Plasmids Carried by Two Nonclonal *bla*_NDM-5_- and *mcr-1*-Bearing Escherichia coli Strains: Insight into Plasmid Transmission among Foodborne Bacteria

**DOI:** 10.1128/Spectrum.00217-21

**Published:** 2021-09-01

**Authors:** Xiaobo Liu, Ruichao Li, Ning Dong, Lianwei Ye, Edward Wai-Chi Chan, Sheng Chen

**Affiliations:** a Shenzhen Key Lab for Food Biological Safety Control, Food Safety and Technology Research Center, Hong Kong PolyU Shen Zhen Research Institute, Shenzhen, People’s Republic of China; b Jiangsu Co-innovation Center for Prevention and Control of Important Animal Infectious Diseases and Zoonoses, College of Veterinary Medicine, Yangzhou University, Yangzhou, People’s Republic of China; c Department of Infectious Diseases and Public Health, Jockey Club College of Veterinary Medicine and Life Sciences, City University of Hong Kong, Kowloon, Hong Kong SAR; d The State Key Lab of Chemical Biology and Drug Discovery, Department of Applied Biology and Chemical Technology, The Hong Kong Polytechnic University, Hung Hom, Kowloon, Hong Kong SAR; University of Manitoba

**Keywords:** foodborne *E. coli*, *bla*_NDM-5_, *mcr-1*, genetic analysis

## Abstract

Our objective was to characterize the genetic features of plasmids harbored by two genetically related, MCR-1 and NDM-5-producing Escherichia coli strains recovered from a chicken meat sample. The genetic profiles of all plasmids harbored by the two test strains, namely, 1106 and 1107, were determined by whole-genome sequencing, S1-pulsed-field gel electrophoresis (PFGE), Southern hybridization, and bioinformatics analysis. The transferability of plasmids harbored by the two strains was assessed by filter mating assay. Strains 1106 and 1107 were resistant to almost all the antibiotics, including colistin and fosfomycin, but remained susceptible to amikacin and tigecycline. The plasmids of p1107-NDM-5 and p1106-NDM-5 both contain a class I integron which lacks the IS*Aba125* element. The backbone of p1106-IncFII exhibited a high degree of similarity with that of p1106-NDM-5 and p1107-NDM-5, implying that events of plasmid fusion and resolution were involved in the formation of the two plasmids. The plasmids p1106-IncHI2MCR and p1107-IncHI2MCR belong to an IncHI2 replicon type, with three copies of IS*Apl1* being observed in p1106-IncHI2MCR, implying that the *mcr-1* gene was transferable among bacteria that reside in the same food matrix. In this study, p1106-IncFIB, p1107-99K, p1107-111K, and p1107-118K were all found to be phage-like plasmids, with p1106-IncFIB and p1107-118K containing several virulence genes, including *iroBCDEN*, *iucABCD*, *sitABCD*, *hlyF*, and *iss*. Surprisingly, resistance genes such as *aph(3′)-Ia*, *sul3*, and *aac(3′)-IId* could also be found in p1107-118K, but resistance genes were not detected in other phage-like plasmids. In conclusion, enhanced surveillance is required to monitor and control the dissemination of various resistance determinants among foodborne pathogens.

**IMPORTANCE** Carbapenem and colistin are last-resort antibiotics used to treat serious clinical infections caused by multidrug-resistant (MDR) bacterial pathogens. Plasmids encoding resistance to carbapenems and colistin have been reported in clinical pathogens in recent years, and yet few studies reported cocarriage of *mcr* and *bla*_NDM_ genes in Escherichia coli strains of food origin. How plasmids encoding these two important resistance determinants are being evolved and transmitted in bacterial pathogens is not well understood. In this study, we investigated the genetic features of plasmids harbored by two nonclonal, *mcr-1*- and *bla*_NDM-5_-bearing E. coli strains (1106 and 1107) recovered from a fresh chicken meat sample to understand and provide evidence of the level and dynamics of MDR plasmid transmission. Our data confirmed that active plasmid fusion and resolution events were involved in the formation of plasmids that harbor multiple resistance genes, which provide insights into the further control of plasmid evolution in bacterial pathogens.

## OBSERVATION

The increasing prevalence of carbapenem-resistant *Enterobacteriaceae* (CRE) strains and dissemination of such strains in clinical settings pose an urgent threat to human health and promote the resurrection of colistin as a last-resort treatment option. The New Delhi metallo-β-lactamase (NDM) is one of the most clinically important carbapenemases due to the fact that the gene encoding this enzyme is located in transferable mobile genetic elements and often coexists with many other resistance determinants (1). Since the discovery of the transmissible colistin resistance gene *mcr-1* in China, numerous descriptions of the MCR homologues (MCR-1 to MCR-10) have been reported in different countries (2). Of particular concern is the inevitable concurrence of *mcr* and *bla*_NDM_ genes in mobile genetic elements harbored by CRE, which resulted in the emergence of pandrug resistance. To date, several studies have reported carriage of *mcr-1* by strains of various species harboring *bla*_NDM-1_, *bla*_NDM-5_, *bla*_NDM-9_, or *bla*_NDM-16_ (3–6). We therefore investigated the scope of the genetic structures of mobile elements underlying the evolutionary changes in *mcr*- and *bla*_NDM_-bearing strains isolated from food samples.

## IDENTIFICATION OF *bla*_NDM-5_/*mcr-1*-BEARING PLASMIDS AND ASSESSMENT OF THEIR TRANSFERABILITY

We collected fresh meat samples, including pork, chicken, beef, and shrimp, from supermarkets and wet markets in Shenzhen, China (supplemental materials and methods). In total, we isolated 1,106 Escherichia coli strains from 2,137 food samples, of which 390 were found to be resistant to colistin and 42 were resistant to meropenem. However, only 5 of the 1,106 strains were found to carry both the *mcr* and *bla*_NDM_ genes in PCR tests, among which two strains, namely, 1106 and 1107, were isolated from the same chicken sample. These two strains were shown to not be genetically related by XbaI-PFGE (see Fig. S1 in the supplemental material) and exhibited resistance to most of the antimicrobial drugs tested, except for amikacin and tigecycline (Table S1). Both carbapenem and colistin resistance phenotypes of E. coli 1106 could be transferred to E. coli strain J53 and produced the transconjugants CTC1106 and MTC1106, respectively; yet, only the carbapenem resistance phenotype of E. coli 1107 could be transferred to E. coli J53 to generate the transconjugant MTC1107. S1-PFGE and Southern blot analysis revealed that six plasmids (∼60 kb, ∼80 kb, ∼90 kb, ∼110 kb, ∼190 kb, and ∼270 kb) were harbored by strain 1106, while strain 1107 was found to carry several plasmids with sizes of over ∼100 kb, as several bands with sizes of ∼110 kb to ∼120 kb and one with a size of ∼230 kb were detectable in S1-PFGE. Southern hybridization showed that the *bla*_NDM-5_ and *mcr-1* genes were located in two plasmids with a size of ∼120 kb and 230 kb, respectively, in this strain (see Fig. S2 in the supplemental material). Moreover, the *mcr-1*-bearing plasmid with a size of 60 kb in strain 1106 was conjugative, while the ∼270-kb *mcr-1*-plasmid in 1106 and the ∼230-kb *mcr-1*-plasmid in 1107 were not conjugative. The MIC of these transconjugants confirmed the transfer of resistance phenotypes (see Table S1 in the supplemental material). Multilocus sequence typing (MLST) was performed on E. coli 1106 and 1107, with results showing that they belonged to sequence type 83 (ST83) and ST21, respectively.

## GENETIC CHARACTERIZATION OF PLASMIDS HARBORING THE *bla*_NDM-1_ GENE IN E. coli STRAINS 1106 AND 1107

**FIG 1:**
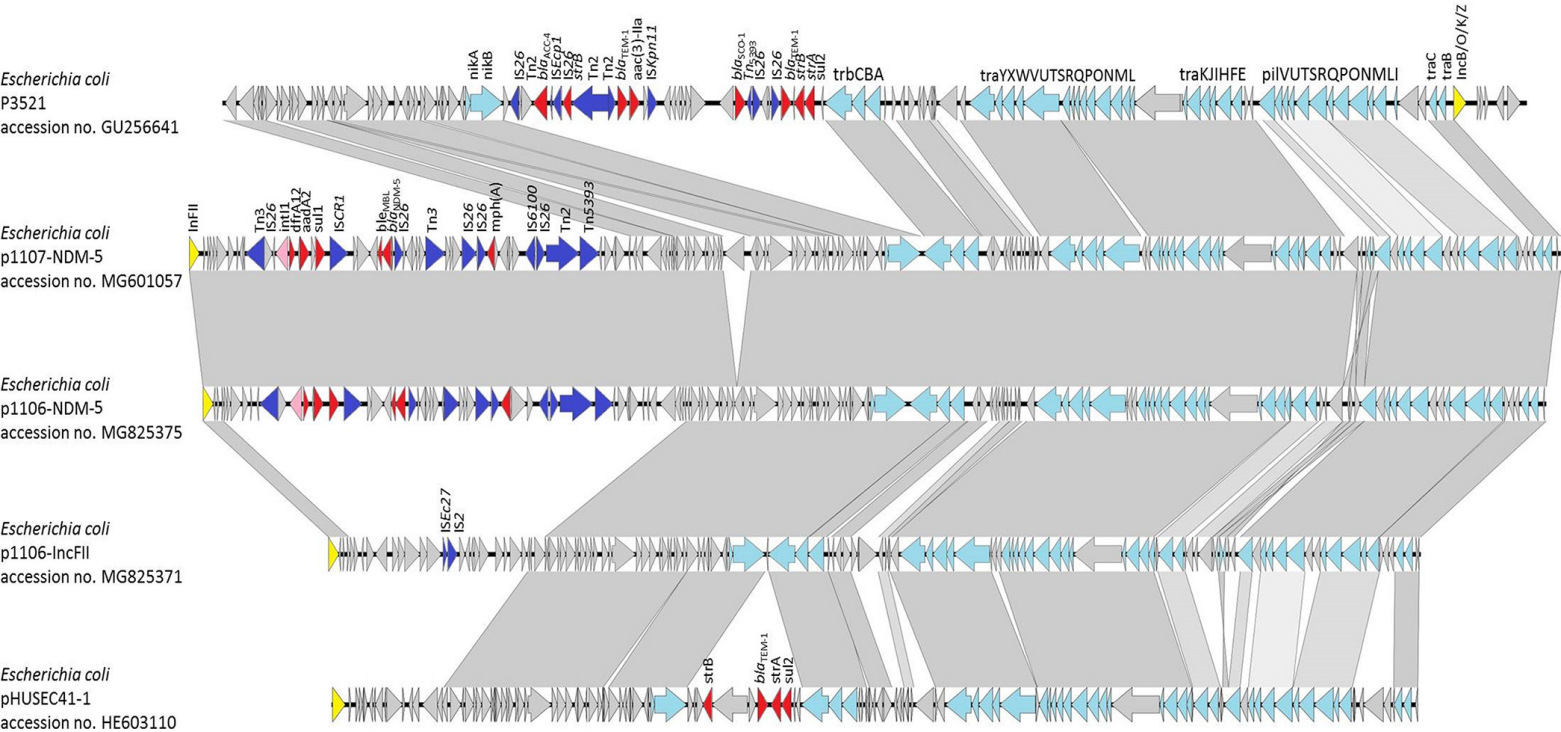
Genetic features of IncFII-type plasmids in E. coli strains 1106 and 1107. Sequence alignments of pHUSEC41-1 (GenBank accession no. HE603110), p3521 (GU256641), p1106-IncFII (MG825371), p1106-NDM-5 (MG825375), and p1107-NDM-5 (MG601057). Light-gray shading denotes shared regions of homology. Gray shading indicates homology between the corresponding genetic loci in each plasmid. Arrows indicate CDSs, with arrowheads indicating the direction of transcription. Red, resistance genes; pink, integrase genes; blue, mobile elements; yellow, replication genes; cyan, genes coding for plasmid transfer; gray, hypothetical proteins or other plasmid scaffold regions.

**FIG 2:**
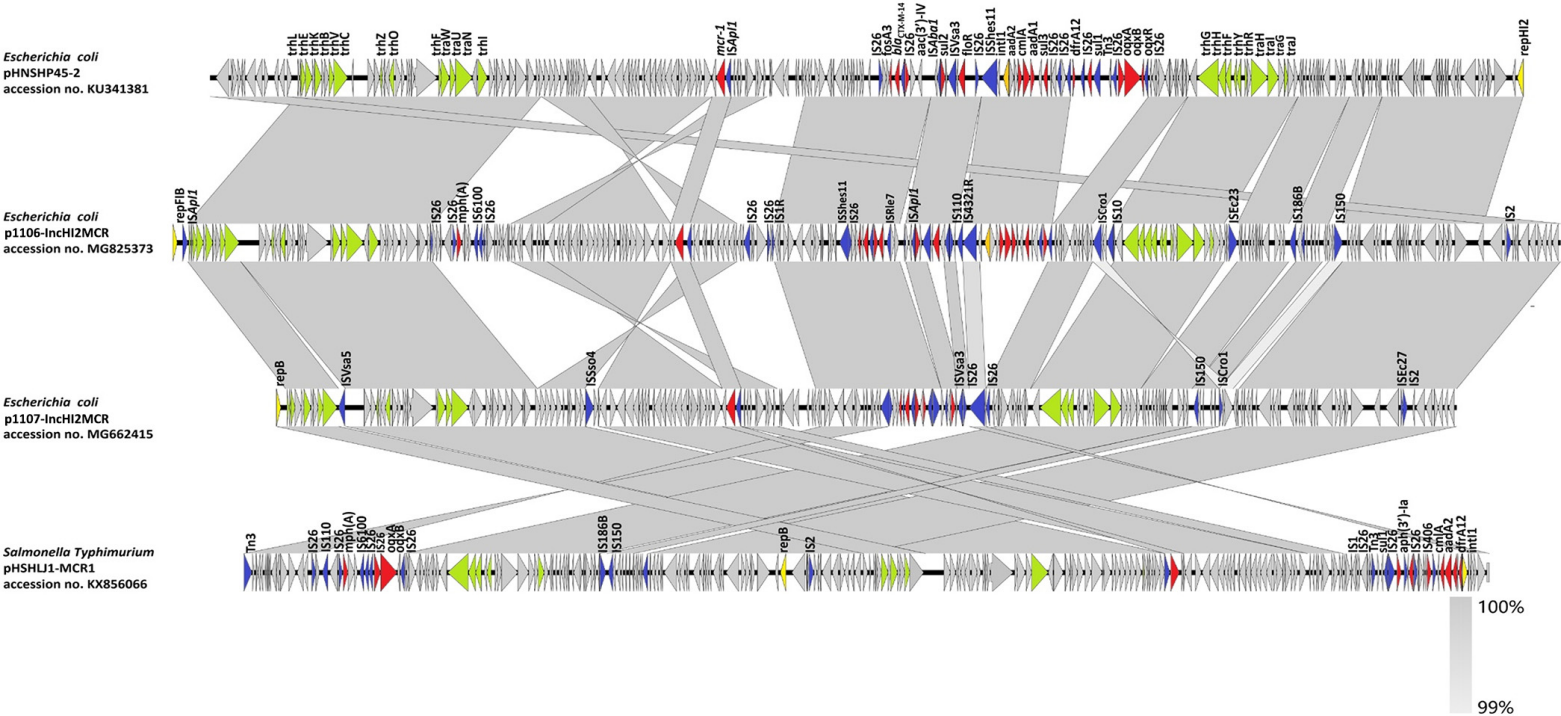
Genetic features of *mcr-1*-bearing plasmid in E. coli strains 1106 and 1107. (A) Sequence alignments of pHNSHP45-2 (GenBank accession no. KU341381), p1106-IncHI2MCR (GenBank accession no. MG825373), p1107-IncHI2MCR (GenBank accession no. MG662415), and pHSHLJ1-MCR1 (GenBank accession no. KX856066). Light-gray shading denotes shared regions of homology. Gray shading indicates homology between the corresponding genetic loci in each plasmid. Arrows indicate CDSs, with arrowheads indicating the direction of transcription. Red, resistance genes; orange, integrase genes; blue, mobile elements; yellow, replication genes; prasinous, genes coding for plasmid transfer; gray, hypothetical proteins or other plasmid scaffold regions.

The complete sequences of plasmids harbored in E. coli strains 1106 and 1107 were decoded by Illumina and Nanopore sequencing and analyzed as described in the supplemental materials and methods and summarized in Table S2 in the supplemental material. The *bla*_NDM-5_-bearing plasmid, from strains 1107 and 1106 and designated p1107-NDM-5 (116042 bp) and p1106-NDM-5 (113687 bp), respectively, were found to contain 161 and 155 protein-coding sequences (CDSs), with a GC content of 54.6% and 54.7%, respectively (Fig. 1). Both p1107-NDM-5 and p1106-NDM-5 belong to the IncFII incompatibility group but contain an additional IncB/O/K/Z replicon gene (see Fig. S3 in the supplemental material). A comparative analysis showed that p1106-NDM-5 aligned well with p1107-NDM-5 but lacked a 2,355-bp region that encodes a retron-type RNA-directed DNA polymerase (Fig. 2, Fig. S3). Comparative analysis revealed that p1106-NDM-5 and p1107-NDM-5 shared extensive structural and sequence similarities with the backbone of the IncI1 plasmid pHUSEC41-1 (HE603110) (7) and an IncB plasmid p3521 (GU256641) (8). However, they lacked an MDR region which contains the *bla*_NDM-5_ gene (Fig. 2).

Intriguingly, the backbone of p1106-NDM-5 and p1107-NDM-5 also exhibited a high degree of structural similarity with that of another plasmid in strain 1106. This plasmid, which belonged to the IncFII incompatibility group with unknown function, was designated p1106-IncFII (92,438 bp). A BLASTN search revealed that p1106-IncFII was also closely related to pMb1536 (KY689635; 79% query coverage and 99% nucleotide identity) and pMb1488 harbored by E. coli strains isolated in Switzerland (KY565558; 80% query coverage and 99% nucleotide identity) (see Fig. S4 in the supplemental material). No AMR gene was detected on this plasmid. Plasmids p1106-NDM-5, p1107-NDM-5, and p1106-IncFII shared the same IncFII replicon and the 75-kb transfer region; however, p1106-IncFII did not harbor the MDR region found in p1106-NDM-5 (Fig. 2), suggesting that formation of p1106-NDM-5 may be attributed to acquisition of an MDR region by the backbone of p1106-IncFII, which resulted in plasmid fusion.

## CHARACTERIZATION OF PLASMIDS HARBORING THE *mcr-1* GENE IN STRAINS 1106 AND 1107

The *mcr-1*-bearing plasmid p1106-IncI2MCR has been described in our previous study (9). The plasmids p1106-IncHI2MCR (MG825373) and p1107-IncHI2MCR (MG662415) both belong to the IncHI2 replicon type and exhibit the core features of the IncHI2 backbone, including having genes responsible for replication, stability, and conjugation. The complete sequences of p1106-IncHI2MCR and p1107-IncHI2MCR displayed 99% nucleotide identity to pXGE1mcr (KY990887) (10), pHNSHP45-2 (KU341381) (11), and pECJS-59-244 (KX084394) (1), which were carried by E. coli strains isolated from fecal samples, and pHSHLJ1-MCR1 (KX856066) that was (12) carried by Salmonella enterica serovar Typhimurium strain HSHLJ1 isolated from human stool (Fig. 2; see Fig. S5 in the supplemental material). Interestingly, the backbone of p1107-IncHI2MCR was almost identical to that of p1106-IncHI2MCR; however, p1107-IncHI2MCR lacked two IS*26*s; an IS*186B* mobile element; the *mph(A)*, *tet(M)*, *floR*, and *aph(3′)-Ia* resistance genes; and the class I integron compared with p1106-IncHI2MCR (Fig. 2). In addition to the IncHI2 *mcr-1*-bearing plasmid found in strain 1106, this strain, as reported in our pervious study, also harbored another IncI2 type of *mcr-1*-bearing plasmid with a size of 60,960 bp and a GC content of 42.3% (9). It was designated p1106-IncI2MCR; BLASTn analysis showed that p1106-IncI2MCR shared 99% sequence identity with pColR644SK1 (MF175188) and pP111 (KY120365) at 100% coverage (see Fig. S6 in the supplemental material). Other plasmids, including one plasmid from strain 1106, designated p1106-IncFIB, and three plasmids from strain 1107 designated p1107-99K, p1107-111K, and p1107-118K were all bacteriophage-like plasmids; details of these plasmids can be found in the supplemental materials.

In conclusion, the current study reports the genetic features of plasmids harbored by two nonclonal MCR-1- and NDM-5-producing E. coli strains recovered from one single chicken meat sample, by using the MinION nanopore sequencing platform. Recovering similar plasmids that contain the *mcr-1* and *bla*_NDM-1_ genes from two distinct isolates in the same food sample suggested that cotransfer of MDR plasmids is a common event among foodborne bacterial pathogens. The emergence of bacterial pathogens that harbor the *bla*_NDM-5_ and *mcr-1* in retail food raises the alarming possibility that such organisms could be transferred readily to humans.
