# Supplementary material for: Complete Genetic Analysis of Plasmids Carried by Two Nonclonal blaNDM-5- and mcr-1-Bearing Escherichia coli Strains: Insight into Plasmid Transmission among Foodborne Bacteria
Source: Microbiol Spectr. 2021 Sep 1;9(2):e00217-21. doi: 10.1128/Spectrum.00217-21 (PMC8557939; doi:10.1128/Spectrum.00217-21)
Supplement: SUPPLEMENTAL FILE 1 — Supplemental material. Download SPECTRUM00217-21_Supp_1_seq5.pdf, PDF file, 1.9 MB. [file spectrum00217-21_supp_1_seq5.pdf]

Supplementary materials

Complete genetic analysis of plasmids carried by two non-clonal *bla*$_{NDM-5}$- and *mcr-1*-bearing *Escherichia coli* strains isolated from a chicken meat sample: insights into plasmid transmission among bacteria on food samples

Xiaobo Liu[1#], Li Ruichao[2#], Ning Dong[3], Lianwei Ye[3], Edward Wai-Chi Chan[4], Sheng Chen[3]*

Running title: Complete genetic analysis of MDR plasmids

[1]Shenzhen Key Lab for Food Biological Safety Control, Food Safety and Technology Research Center, Hong Kong PolyU Shen Zhen Research Institute, Shenzhen, P. R. China;

[2]Jiangsu Co-innovation Center for Prevention and Control of Important Animal Infectious Diseases and Zoonoses, College of Veterinary Medicine, Yangzhou University, Yangzhou, P. R. China;

[3]Department of Infectious Diseases and Public Health, Jockey Club College of Veterinary Medicine and Life Sciences, City University of Hong Kong, Kowloon, Hong Kong SAR;

[4]The State Key Lab of Chemical Biology and Drug Discovery, Department of Applied Biology and Chemical Technology, The Hong Kong Polytechnic University, Hung Hom, Kowloon, Hong Kong SAR.

[#]These authors contributed equally to this work.

*Corresponding authors:

Sheng CHEN, Tel: 852-34425782, Email: shechen@cityu.edu.hk

Keywords: Foodborne E. coli, blaNDM-5, mcr-1, Copy repeats, Genetic analysis

Supplementary materials and methods

Bacterial isolation

E. coli isolates 1106 and 1107 were recovered from a chicken meat sample purchased from a supermarket in Shenzhen, Guangdong Province, China in March 27th, 2017. However, they were obtained in two different ways for the reason described as follows. In our previous studies on isolation of *Salmonella* from meat samples, we found that some of the colonies picked up from XLT4 plates were *E. coli*. These *E. coli* isolates were phenotypically very different from *E. coli* isolates that we isolated from the same meat products using MacConkey agar plates. Although we did not understand the underlying reason for discrepancy of colony morphology in *E. coli* isolated by the two methods, we intended to simultaneously use these two methods for isolation of *E. coli* during our surveillance. In brief, 25 g of chicken meat sample were placed in a sterile homogeneous bag containing 50 ml of sterilized saline. Strain 1106 was obtained after a loopful of food suspension was spread onto MacConkey agar plates supplemented with 0.5μg/ml meropenem, followed by incubation at 37℃ overnight. Strain 1107 was obtained by the following procedure. Briefly, 1ml of homogenate was transferred to Lactose broth which was then incubated at 42℃ for 12-16 h. One milliliter of this pre-enriched broth was transferred to Rappaport-Vassiliadis broth (RV broth) and incubated overnight at 37℃. A loopful of the culture was inoculated onto the XLT4 agar plate supplemented with 0.5μg/ml meropenem. After incubation at 37℃ overnight, a colony with typical *E. coli* morphology was selected. Both 1106 and 1107 were identified to be *E. coli* by MALDI-TOF MS using a Bruker MicroFlex LT mass spectrometer (Bruker Daltonics) and API20E test strip (BioMerieux, Inc).

Antimicrobial susceptibility tests

Antimicrobial susceptibility tests for strain 1106 and 1107 were performed using the agar dilution method according to the Clinical and Laboratory Standards Institute (CLSI)(1). Antibiotics used were listed in **Table 1**. Resistance breakpoints were interpreted according to CLSI recommendations. *E. coli* strain ATCC 25922 was used as the quality control strain.

Conjugation, S1-PFGE and Southern hybridization

The transferability of resistance phenotypes was confirmed by filter mating assays performed as previously described, with slight modification(2). Briefly, cultures of donor strains (*E. coli* 1106 and 1107) and the recipient strain (sodium-azide-resistant *E. coli* strain J53) were mixed at a ratio of 4:1, inoculated onto a filter membrane which was then placed on LB agar medium without antibiotics, and incubated at 37°C for 16 hours. Transconjugants were selected on MacConkey agar plates supplemented with meropenem (1μg/mL) and sodium azide (200μg/mL) or on Eosin Methylene Blue Agar containing sodium azide (100μg/mL) and colistin (2μg/mL). The genetic relatedness of strain 1106 and 1107 were determined by PFGE using the Chef-Mapper pulsed-field electrophoresis system (Bio-Rad, USA) upon XbaI digestion. The BioNumerics (Applied Maths) system was used to perform cluster analysis of PFGE patterns. S1-nuclease PFGE was performed to characterize the plasmids that conferred resistance to meropenem and colistin. The genetic location of $bla_{\text{NDM-5}}$ and *mcr-1* in strain 1106 and 1107 and their corresponding transconjugants were identified by Southern hybridization, using $bla_{\text{NDM-1}}$ and *mcr-1* digoxigenin-labeled probes in accordance with the instructions of the

DIG-High Prime DNA Labeling and Detection Starter Kit II (Roche Diagnostics). The genomic DNA of strain 1106 and 1107 were sequenced and their multilocus sequence types (MLST) were assigned using online databases (http://bigsdb.pasteur.fr/) for *E. coli*.

Plasmid sequencing and bioinformatics analyses

To determine the complete nucleotide sequences of the plasmids harbored by *E. coli* 1106 and 1107, plasmids were extracted from *E. coli* strain 1106 and 1107 by using the QIAGEN Plasmid Midi Kit (Qiagen, Valencia, CA). The quality of plasmid DNA was measured by Nanodrop and subjected to short-read and long-read sequencing of Illumina Nextseq 500 and ONT MinION sequencers to obtain accurate complete plasmid sequences. Library preparation and data analysis were performed according to published methods with minor modification(3). The sequence accuracy of raw Nanopore read is below 97%, and consensus accuracy of assembly with high sequencing coverage could reach up to 99%, but still did not meet the high-quality genome sequences requirements. We therefore used hybrid assembly strategy combining short-read Illumina data and long-read Nanopore data with Unicycler (v0.4.4) to generate high-quality sequences. In Unicycler, the Pilon tool was used to correct assemblies with accurate short-read Illumina data. This quality control steps could be retrieved from the software manual (4). Alignment of MDR plasmids and nanopore long reads were visualized by the Easyfig and BRIG tools(5, 6). All complete sequences were submitted to NCBI GenBank database with accession numbers as listed in **Table 2**.

Supplementary results

Apart from the plasmids described above in these two *E. coli* strains, strain 1106 also harbored another plasmid and 1107 harbored three more plasmids. One plasmid from strain 1106, designated as p1106-IncFIB, and three plasmids from strain 1107 designated as p1107-99K, p1107-111K and p1107-118K respectively, were all bacteriophage like plasmids. Bacteriophage, one of mobile genetic elements involved in horizontal gene transfer in microorganisms, could be integrated into the chromosome or transmitted to the new host through self-replication(7). Plasmid p1106-IncFIB was 190,401bp in size, with a GC content of 49.8%. It exhibits 99% nucleotide similarity with two ColV plasmids including the plasmid p1ColV5155(CP005931)(8) carried by *E. coli* strain IMT5155 at 90% coverage and pAPEC-O1-ColBM (DQ381420)(9) carried by an avian pathogenic *E. coli* (APEC) strain O1 at 63% coverage (**Fig. S6**). The plasmid comprises a virulence region and a large transfer region; the virulence region contains several virulence genes associated with APEC strains, including the *iroBCDEN* genes encoding the siderophore receptor, the *iucABCD* and *iutA* genes of the aerobactin iron transport system, the *sitABCD* genes of the manganese ABC transport system, and the *hlyF* and *iss* genes of the salmochelin operon. Such structure was similar to those of p1ColV5155 and pAPEC-O1-ColBM(8, 9) (**Fig. S6**). However, p1106-IncFIB lacked the *cvaA* and *cvaB* genes of ColV operon, indicating that p1106-IncFIB might not belong to ColV plasmids. On the other hand, the 32kb transfer region of p1106-IncFIB was also structurally similar to a phage-harboring MDR plasmid, pKP12226 (KP453775), which was carried by a *K. pneumoniae* strain isolated in South Korea(10)(**Fig. S6**).

BLASTN analysis revealed that p1107-99K exhibited the highest similarity with the plasmid

RCS47 harbored by an *E. coli* strain isolated in Paris, and that both plasmids belonged to an unknown incompatibility group. RCS47 was found to harbor a *bla*$_{SHV-2}$ gene(11), however, no resistance genes were detected in p1107-99K, of which 81% of the sequence was identical to P1 bacteriophage (AF234172)(12) (**Fig. S7**). The sequence of plasmid p1107-111K was similar to that of the *E. coli* plasmid LF82 (CU638872)(13), which was associated with Crohn's disease and at a high query coverage of 89% and a bacteriophage-like plasmid pECOH89(HG530657)(14) from an *E. coli* strain containing the IS*Ecp1-bla*$_{CTX-M-15}$ transposition unit at 84% coverage (**Fig. S8**). Nevertheless, p1107-118K exhibited high sequence similarity to an IncHI2 plasmid pP2-3T (MG014722)(15) and an IncFII plasmid pSMS35_130 (CP000971)(16). A large region of ~35kb that contained representative virulence genes including *sitABCD, iroBCDEN, ompT*, *hlyF*, *colicinM* and *iss* genes was observed, in which the corresponding gene clusters are also located in pP2-3T. Worryingly, some resistance genes such as *aph(3')-Ia*, *sul3* and *aac(3')-IId* were also found in p1107-118K (**Fig. S9**). It was noteworthy that phage-like sequences are more commonly reported in *E. coli* and *Acinetobacter baumannii* strains(10, 17), thus dissemination of resistance genes-bearing phage-like plasmids among various bacterial pathogens is a new concern.

Supplementary Table S1. MICs of strain 1106, 1107 and their corresponding transconjugants.

Strains	Antibiotics												
	TIG	CLS	MRP	CAZ/AVB	FOS	KAN	CHL	NAL	AMK	CIP	CTX	AMP	SXT
1106	0.25	4	⩾16	32	⩾512	⩾256	⩾128	⩾128	2	16	⩾32	⩾128	⩾64
CTC1106	0.12	4	0.12	0.12	4	1	4	4	0.5	1	0.25	⩾128	8
MTC1106	0.25	0.5	⩾16	64	4	1	4	2	0.5	0.015	⩾32	⩾128	32
1107	0.25	8	8	32	⩾512	⩾256	64	⩾128	2	16	⩾32	⩾128	32
MTC1107	0.12	0.5	4	⩾128	4	1	4	4	0.5	1	⩾32	⩾128	32

TIG, tigecycline; CLS, colistin; MRP, meropenem; CAZ/AVB, ceftazidime/Avibactam; FOS, fosfomycin; KAN, kanamycin; CHL, chloramphenicol; NAL, nalidixic acid; AMK, amikacin; CIP, ciprofloxacin; CTX, cefotaxime; AMP, ampicillin; SXT, trimethoprim-sulfamethoxazole.

Supplementary Table S2. Genetic features of 10 plasmids identified in *E. coli* strain 1106 and 1107.

Plasmid	Size	G+C	Inc type	Antimicrobial resistance genes	IS elements or transposons	Accession
p1106-IncI2MCR	60,960	42.3	IncI2	*mcr-1*	NT	MG825374
p1106-IncFII	92,438	51.9	IncFII	NT	IS*Ec27*, IS*Cfr3*, IS*2*	MG825371
p1106-NDM-5	113,687	54.7	IncFII	*dfrA12*, *aadA2*, *sul1*, *ble*$_{\text{MBL}}$,*bla*$_{\text{NDM-}}$ [illegible]	Tn*2*, IS*26*, IS*CR1*, IS*6100*, Tn*2*, Tn*5393*	MG825375
p1106-IncFIB	190,401	49.8	IncFIB	NT	IS*5*, IS*Cro3*, IS*1595*, IS*2*, IS*Ec27*, IS*Kpn28*, IS*3*, IS*Ec8*, IS*30*, IS*91*, IS*110*, IS*1*, Tn*3*, IS*Ec38*, IS*21*	MG825372
p1106-IncHI2MCR	265,799	46.6	IncHI2	*mph(A)*,*mcr-1*,*tet(M)*,*bla*$_{\text{CTX-M-14}}$, *fosA3*, *aph(4)-Ia*, *sul2*, *floR*, *aadA1*, *aadA2*, *cmlA1*, *sul3*, *aph(3')-Ia*	IS*Apl1*, IS*26*, IS*6100*, IS*1R*, IS*Ecp1*, Tn*3*, IS*Ec59*, IS*Aba1*, IS*Vsa3*, IS*1006*, Tn*5393*, IS*4321R*, IS*Cro1*, IS*10L*, IS*Ec23*, IS*186B*, IS*150*, IS*2*	MG825373
p1107-99K	99,453	47.7	NT	NT	IS*Pa38*, IS*1294*	MH580301
p1107-111K	111,455	46.3	IncFIB	NT	IS*1294*	MG825385
p1107-NDM-5	116,042	54.6	IncFII	*dfrA12*,*aadA2*,*sul1*,*ble*$_{\text{MBL}}$, *bla*$_{\text{NDM-5}}$, [illegible]	Tn*3*, IS*CR1*, IS*26*, Tn*2*, IS*6100*, Tn*5393*	MG601057
p1107-118K	118,160	49.2	IncFIB	*aph(3')-Ia*, *sul3*,*aac(3')-IIc*	IS*Ec32*, IS*1203*, IS*2*, IS*Sso4*, IS*26*, IS*Vsa5*, Tn*2*,	MH580302
p1107-IncHI2MCR	226,105	45.7	IncHI2	*mcr-1*, *fosA3*, *bla*$_{\text{CTX-M-14}}$, *aac(3)-IVa*, *aph(4)-Ia*, *sul2*	IS*Vsa5*,IS*Sso4*, IS*Apl1*, Tn*3*, IS*26*, IS*Ecp1*, IS*Ec59*, Tn*5393*, IS*Aba1*, IS*Vsa3*, IS*150*, IS*Cro1*, IS*Ec27*, IS*2*	MG662415

[a]NT, not detected.

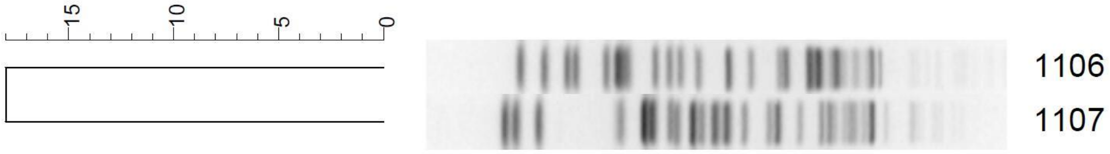

Supplementary Figure S1. XbaI-PFGE pattern of *E. coli* 1106 and 1107.

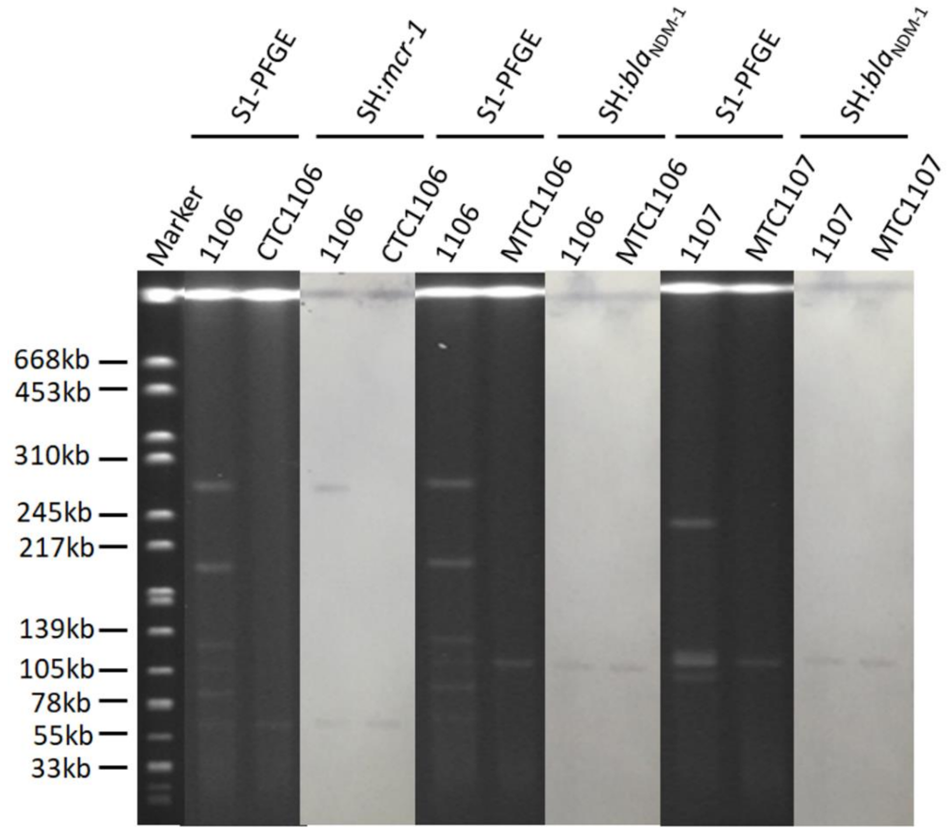

Supplementary Figure S2. S1-PFGE and Southern hybridization analysis of *E. coli* strains 1106 and 1107, and their transconjugants. CTC, transconjugants selected by colistin and sodium azide; MTC, transconjugants selected by meropenem and sodium azide; SH, Southern hybridization.

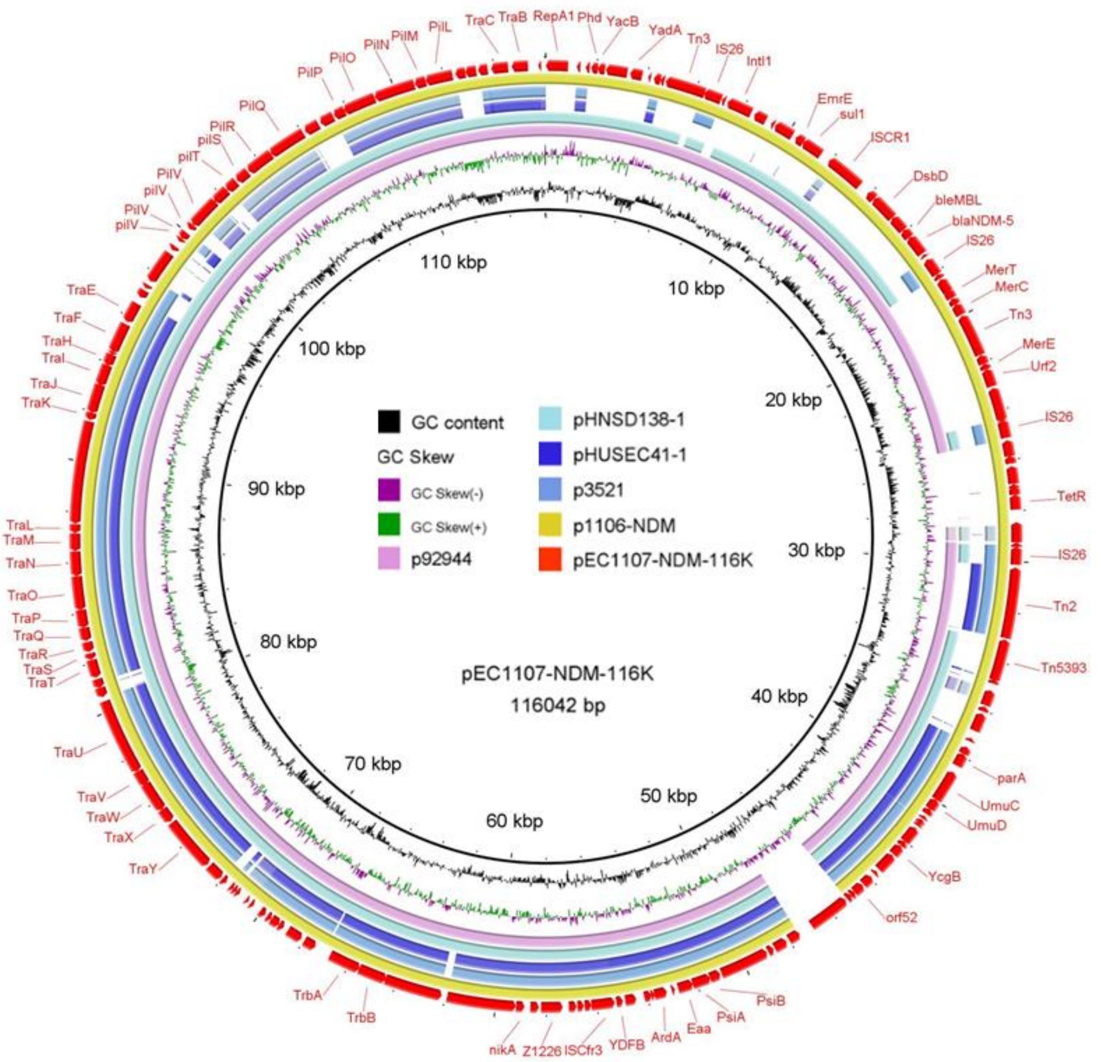

Supplementary Figure S3. Sequence alignment of plasmids p1106-NDM(MG825375), p1107-NDM(MG601057), p92944(MG838206), pHNSD138-1(MG271839), pHUSEC41-1 (HE603110) and p3521(GU256641). The outer circle with red arrows signifies annotation of the reference sequence. Gaps in the circle refer to plasmid regions which are missing compared to the reference.

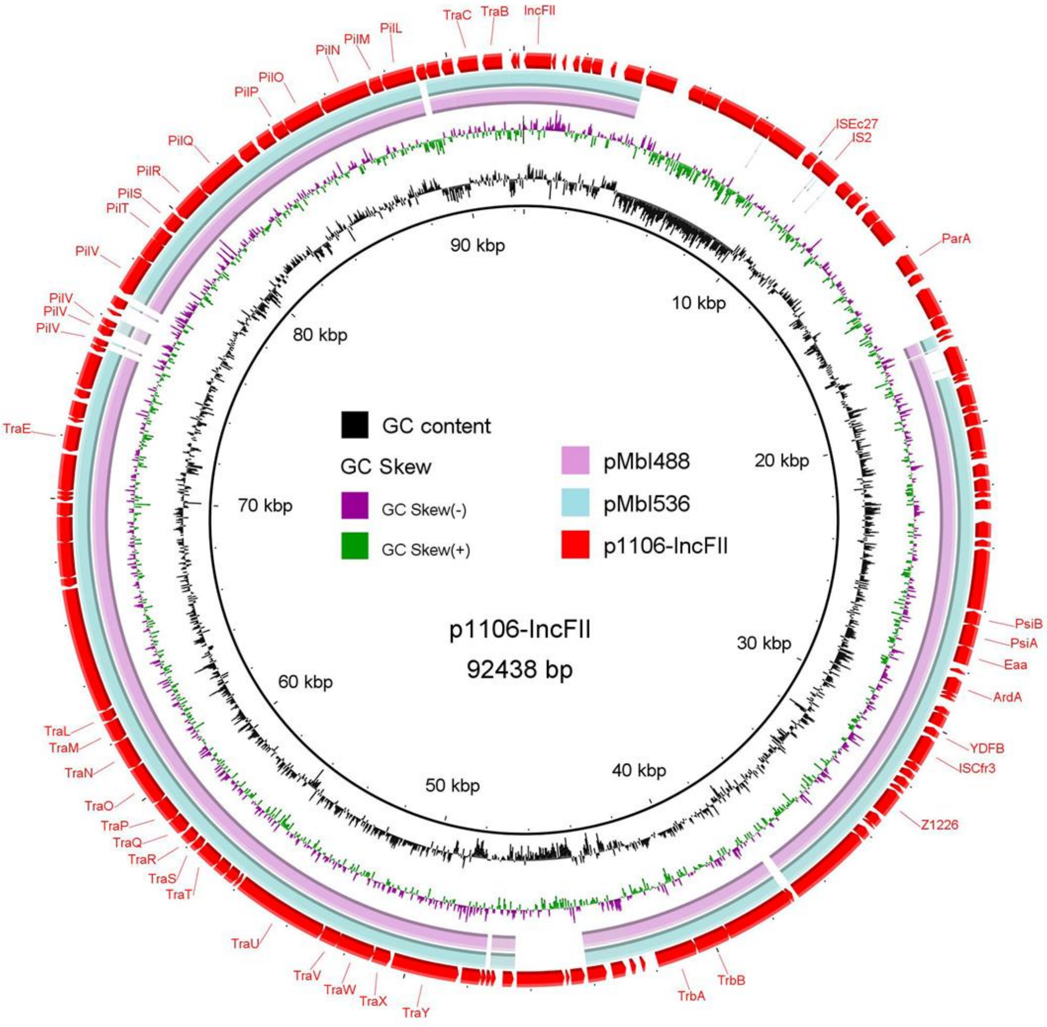

Supplementary Figure S4. Sequence alignment of plasmids p1106-IncFII(MG825371), pMb1488(KY565558) and pMb1536 (KY689635). The outer circle with red arrows signifies annotation of the reference sequence. Gaps in the circle refer to plasmid regions which are missing compared to the reference.

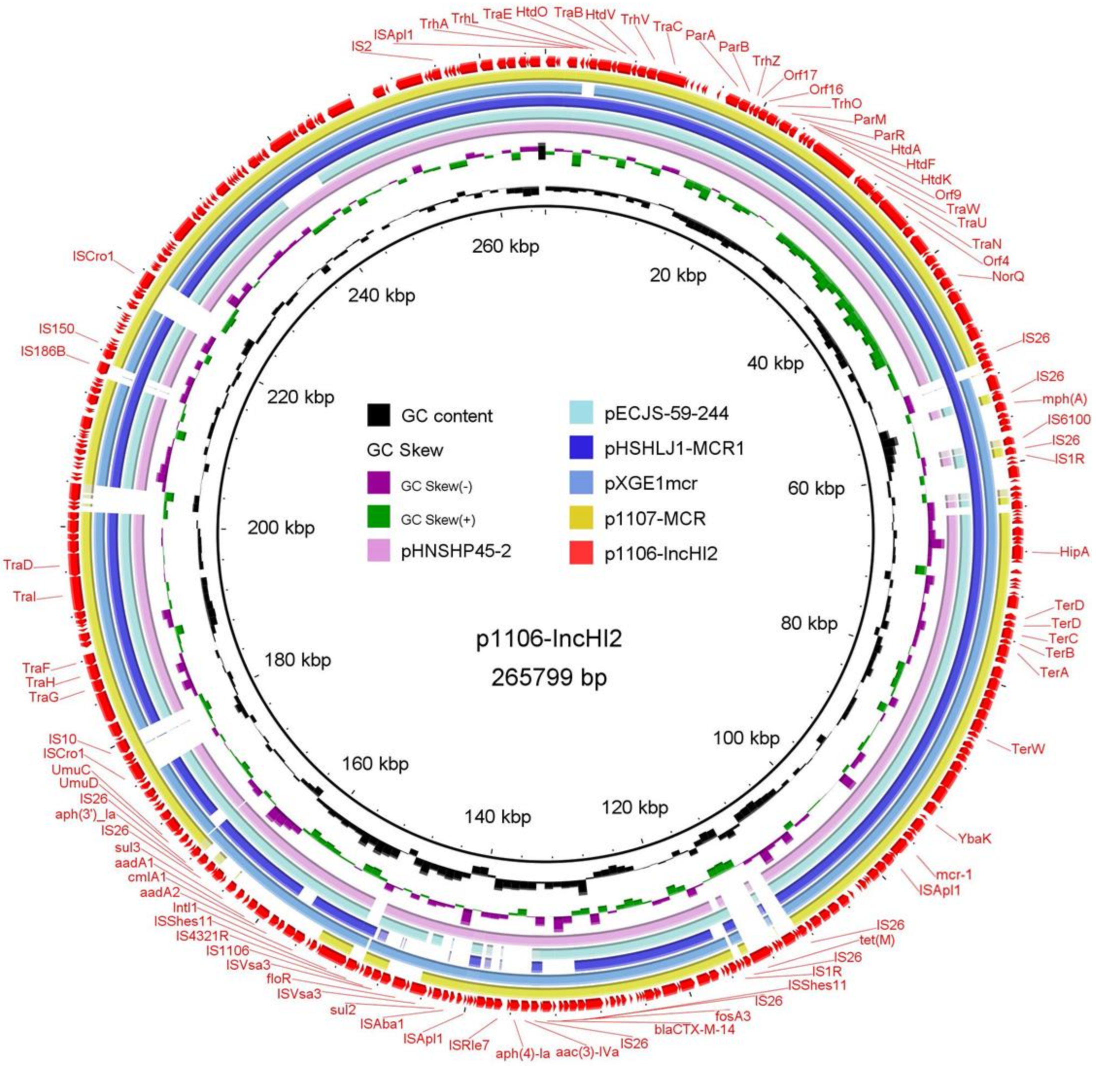

Supplementary Figure S5. Sequence alignment of plasmids p1106-MCR(MG825373), p1107-MCR (MG662415), pHNSHP45-2 (KU341381), pECJS-59-244 (KX084394), pHSHLJ1-MCR1(KX856066) and pXGE1mcr (KY990887). The outer circle with red arrows signifies annotation of the reference sequence. Gaps in the circle refer to plasmid regions which are missing compared to the reference.

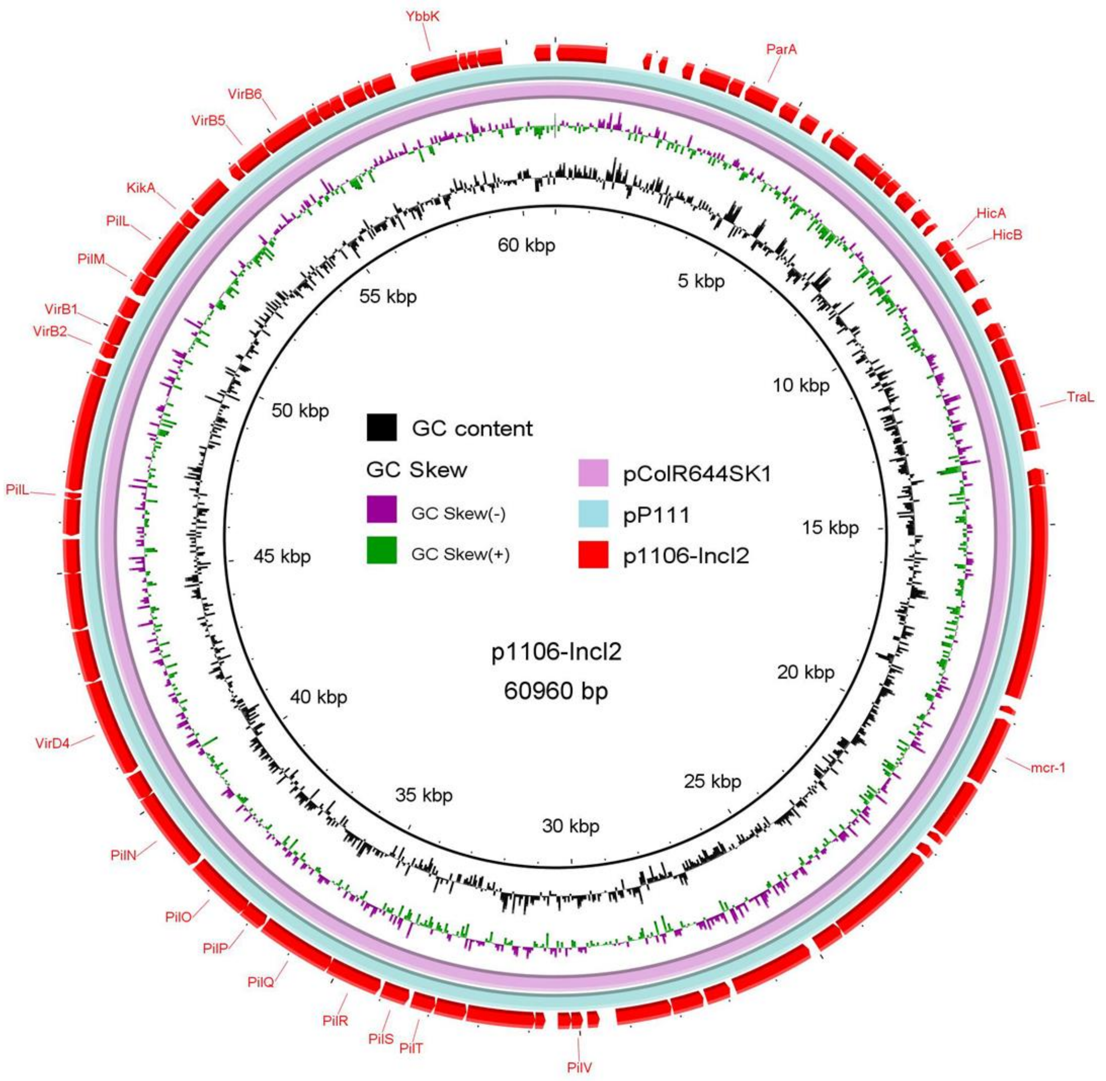

Supplementary Figure S6. Sequence alignment of plasmids p1106-IncI2(MG825374), pColR644SK1(MF175188) and pP111(KY120365). The outer circle with red arrows signifies annotation of the reference sequence. Gaps in the circle refer to plasmid regions which are missing compared to the reference.

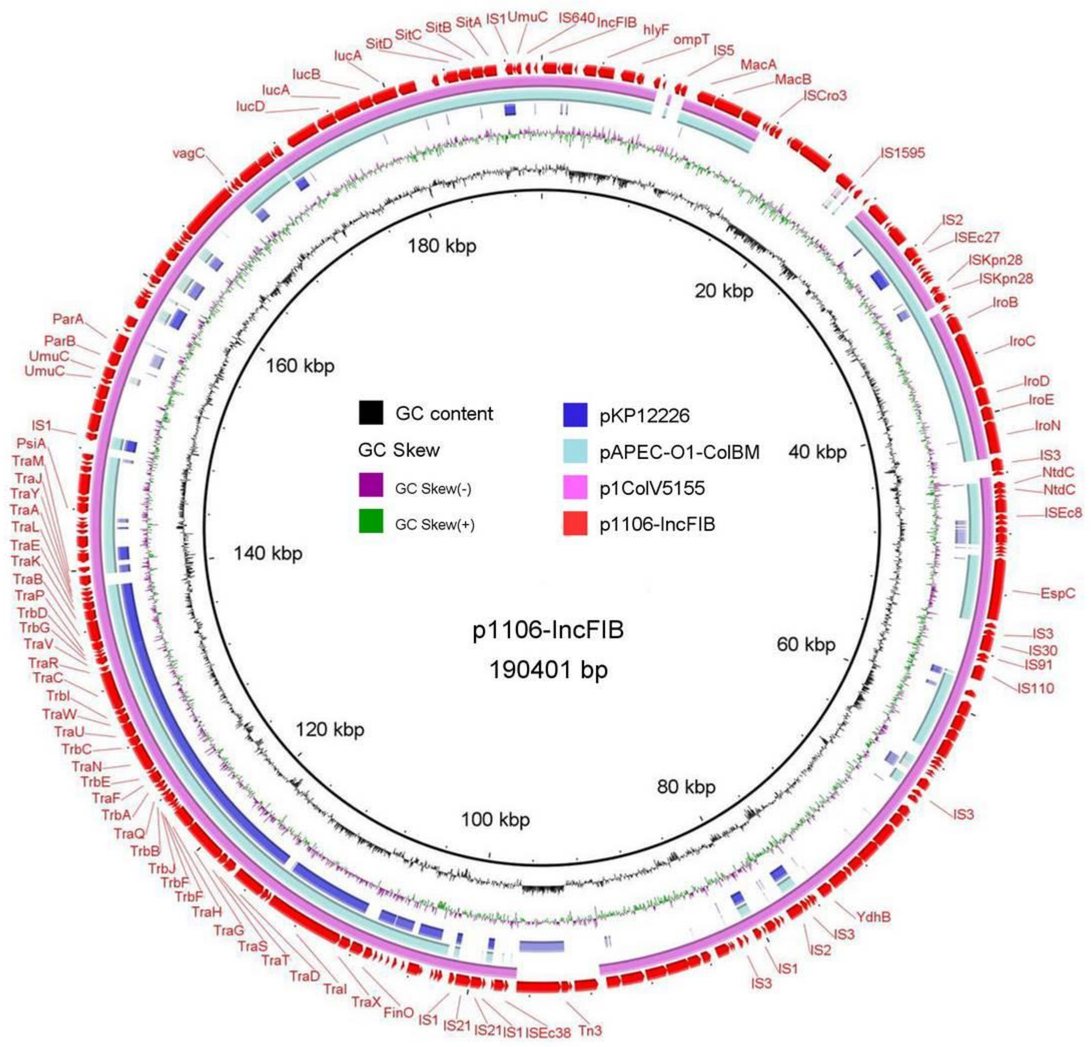

Supplementary Figure S7. Sequence alignment of plasmids p1106-IncFIB (MG825372), pKP12226 (KP453775), pAPEC-O1-ColBM (DQ381420) and p1ColV5155 (CP005931). The latter one was used as a reference. The outer circle with red arrows denotes annotation of the reference sequence, the gaps represent sequences that were found to be missing when compared to the reference plasmid.

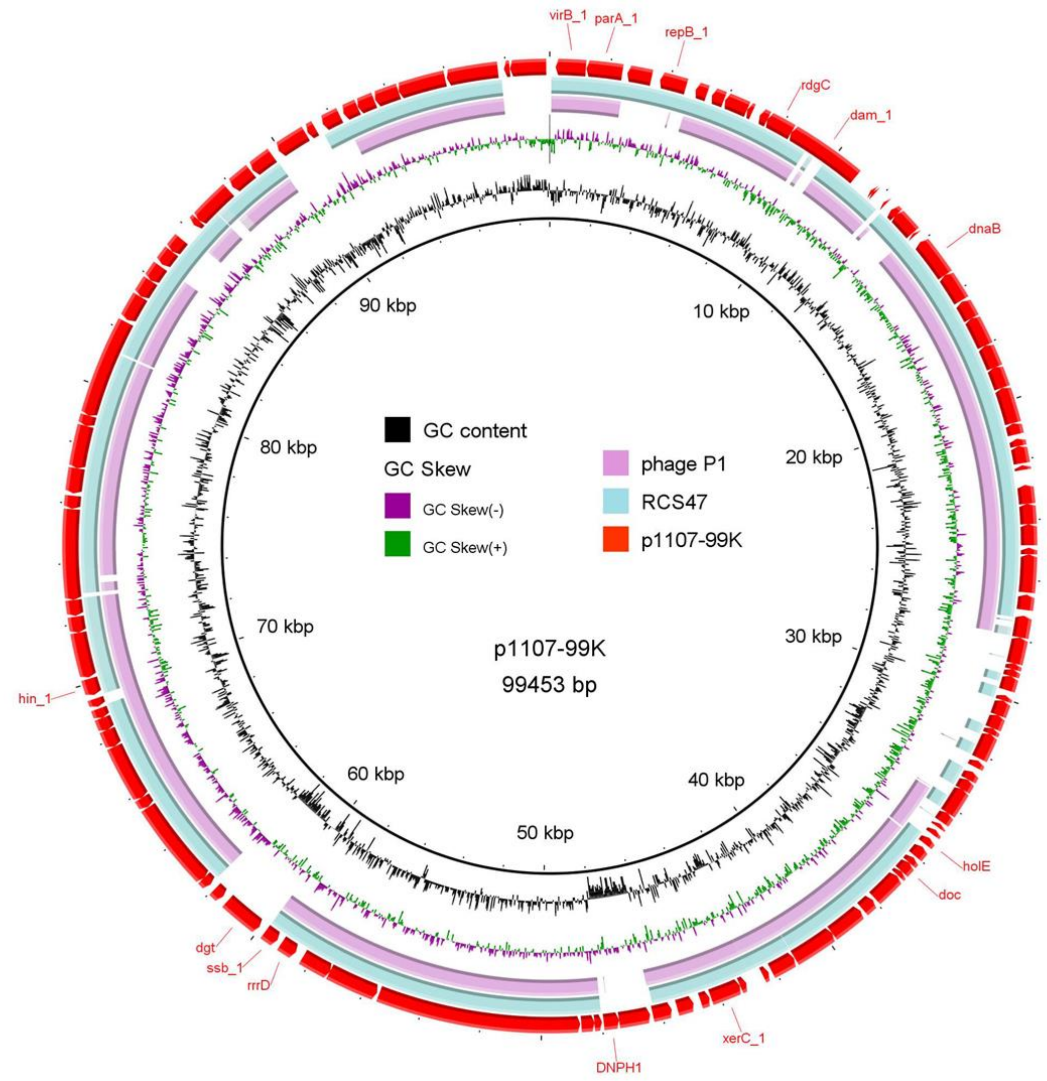

Supplementary Figure S8. Sequence alignment of plasmids p1107-99K(MH580301), P1 phage (AF234172) and RCS47(FO818745). The outer circle with red arrows signifies annotation of the reference sequence. Gaps in the circle refer to plasmid regions which are missing compared to the reference.

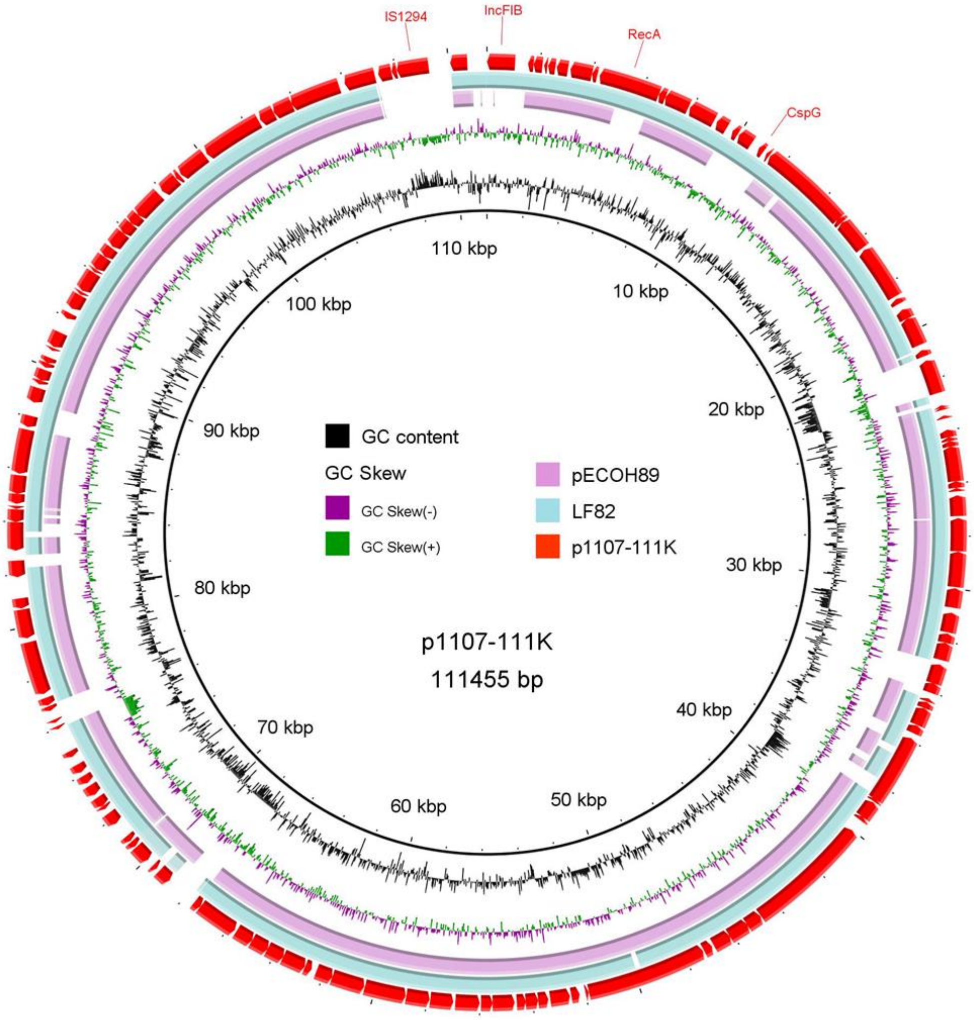

Supplementary Figure S9. Sequence alignment of plasmids p1107-111K(MG825385), pECOH89 (HG530657) and LF82(CU638872). The outer circle with red arrows signifies annotation of the reference sequence. Gaps in the circle refer to plasmid regions which are missing compared to the reference.

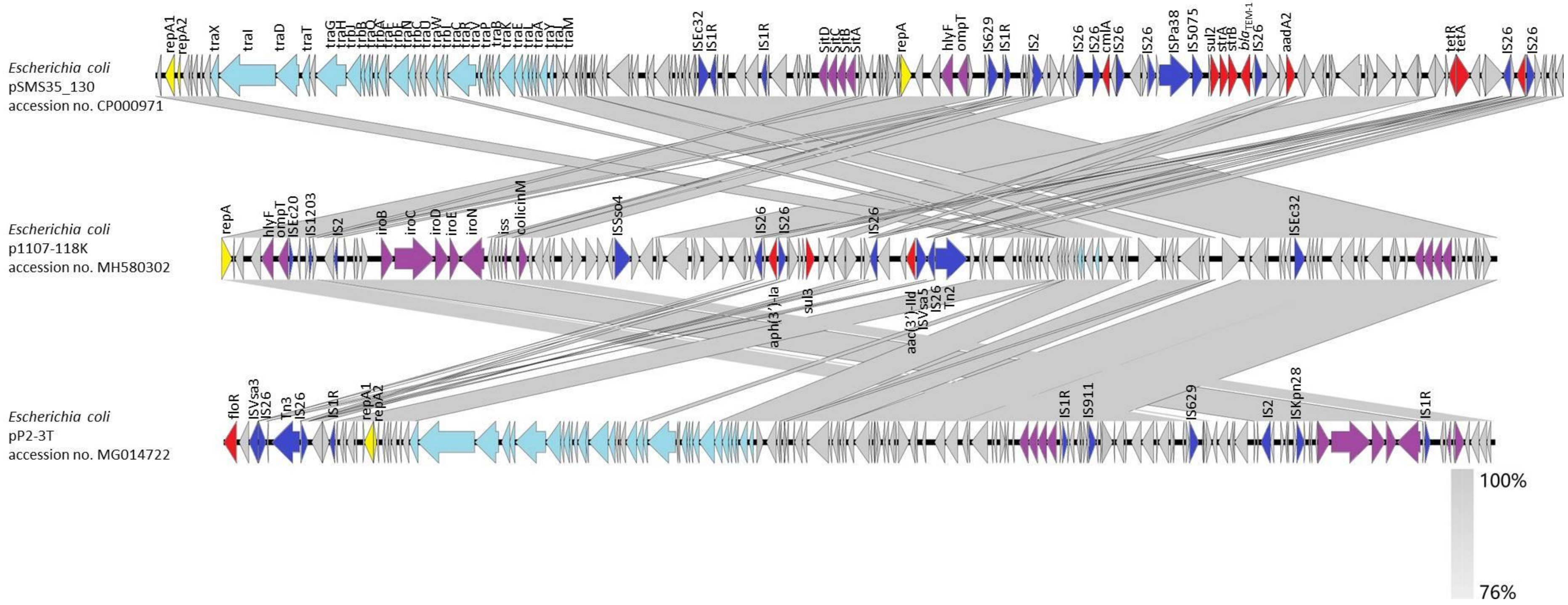

Supplementary Figure S10. Sequence alignment of p1107-118K (MH580302), pSMS35_130 (CP000971) and pP2-3T(MG014722). Light gray shading denotes shared regions of homology. Gray shading indicates homologies between the corresponding genetic loci in each plasmid. Arrows indicate CDSs, with arrowheads indicating the direction of transcription. purple, virulence genes; red, resistance genes; blue, mobile elements; yellow, replication genes; cyan, genes coding for plasmid transfer; gray, hypothetical proteins or other plasmid scaffold regions.

References:

1. CLSI. 2017. Performance Standards for Antimicrobial Susceptibility Testing； Twenty-third informational supplement. CLSI document M100-S27, Wayne, PA: Clinical and Laboratory Standards Institute.
2. Li R, Lin D, Chen K, Wong MH, Chen S. 2015. First detection of AmpC β-lactamase bla(CMY-2) on a conjugative IncA/C plasmid in a Vibrio parahaemolyticus isolate of food origin. Antimicrob Agents Chemother 59:4106-11.
3. Li R, Xie M, Dong N, Lin D, Yang X, Wong MHY, Chan EW, Chen S. 2018. Efficient generation of complete sequences of MDR-encoding plasmids by rapid assembly of MinION barcoding sequencing data. Gigascience 7:1-9.
4. Wick RR, Judd LM, Gorrie CL, Holt KE. 2017. Unicycler: Resolving bacterial genome assemblies from short and long sequencing reads. PLoS Comput Biol 13:e1005595.
5. Sullivan MJ, Petty NK, Beatson SA. 2011. Easyfig: a genome comparison visualizer. Bioinformatics 27:1009-1010.
6. Alikhan NF, Petty NK, Ben Zakour NL, Beatson SA. 2011. BLAST Ring Image Generator (BRIG): simple prokaryote genome comparisons. BMC Genomics 12:402.
7. Croxen MA, Law RJ, Scholz R, Keeney KM, Wlodarska M, Finlay BB. 2013. Recent advances in understanding enteric pathogenic *Escherichia coli*. Clin Microbiol Rev 26:822-80.
8. Zhu Ge X, Jiang J, Pan Z, Hu L, Wang S, Wang H, Leung FC, Dai J, Fan H. 2014. Comparative genomic analysis shows that avian pathogenic *Escherichia coli* isolate IMT5155 (O2:K1:H5; ST complex 95, ST140) shares close relationship with ST95 APEC O1:K1 and human ExPEC O18:K1 strains. PLoS One 9:e112048.
9. Johnson TJ, Johnson SJ, Nolan LK. 2006. Complete DNA sequence of a ColBM plasmid from avian pathogenic *Escherichia coli* suggests that it evolved from closely related ColV virulence plasmids. J Bacteriol 188:5975-83.
10. Shin J, Ko KS. 2015. A Plasmid Bearing the $bla_{\text{CTX-M-15}}$ Gene and Phage P1-Like Sequences from a Sequence Type 11 *Klebsiella pneumoniae* Isolate. Antimicrob Agents Chemother 59:6608-10.
11. Billard-Pomares T, Fouteau S, Jacquet ME, Roche D, Barbe V, Castellanos M, Bouet JY, Cruveiller S, Medigue C, Blanco J, Clermont O, Denamur E, Branger C. 2014. Characterization of a P1-like bacteriophage carrying an SHV-2 extended-spectrum beta-lactamase from an *Escherichia coli* strain. Antimicrob Agents Chemother 58:6550-7.
12. Lobocka MB, Rose DJ, Plunkett G, 3rd, Rusin M, Samojedny A, Lehnherr H, Yarmolinsky MB, Blattner FR. 2004. Genome of bacteriophage P1. J Bacteriol 186:7032-68.
13. Miquel S, Peyretaillade E, Claret L, de Vallee A, Dossat C, Vacherie B, Zineb el H, Segurens B, Barbe V, Sauvanet P, Neut C, Colombel JF, Medigue C, Mojica FJ, Peyret P, Bonnet R, Darfeuille-Michaud A. 2010. Complete genome sequence of Crohn's disease-associated adherent-invasive *Escherichia coli* strain LF82. PLoS One 5.

14. Falgenhauer L, Yao Y, Fritzenwanker M, Schmiedel J, Imirzalioglu C, Chakraborty T. 2014. Complete Genome Sequence of Phage-Like Plasmid pECOH89, Encoding CTX-M-15. Genome Announc 2.
15. Fang LX, Li XP, Deng GH, Li SM, Yang RS, Wu ZW, Liao XP, Sun J, Liu YH. 2018. High Genetic Plasticity in Multidrug-Resistant Sequence Type 3-IncHI2 Plasmids Revealed by Sequence Comparison and Phylogenetic Analysis. Antimicrob Agents Chemother 62.
16. Fricke WF, Wright MS, Lindell AH, Harkins DM, Baker-Austin C, Ravel J, Stepanauskas R. 2008. Insights into the environmental resistance gene pool from the genome sequence of the multidrug-resistant environmental isolate *Escherichia coli* SMS-3-5. J Bacteriol 190:6779-94.
17. Li R, Xie M, Lv J, Wai-Chi Chan E, Chen S. 2017. Complete genetic analysis of plasmids carrying mcr-1 and other resistance genes in an Escherichia coli isolate of animal origin. J Antimicrob Chemother 72:696-699.
